# Phenotypic Differences in a *PRPH2* Mutation in Members of the Same Family Assessed with OCT and OCTA

**DOI:** 10.3390/diagnostics11050777

**Published:** 2021-04-26

**Authors:** Henar Albertos-Arranz, Xavier Sánchez-Sáez, Natalia Martínez-Gil, Isabel Pinilla, Rosa M. Coco-Martin, Jesús Delgado, Nicolás Cuenca

**Affiliations:** 1 Department of Physiology, Genetics and Microbiology, University of Alicante, 03690 Alicante, Spain; henar.albertos@ua.es (H.A.-A.); xsanchez@ua.es (X.S.-S.); natalia.martinez.gil@ua.es (N.M.-G.); 2 Department of Ophthalmology, Aragon Health Science Institute (IIS Aragón), Lozano Blesa University Hospital, 50009 Zaragoza, Spain; ipinilla@unizar.es; 3 National Institute of Health Carlos III (ISCIII), (RETICS) Cooperative Health Network for Research in Ophthalmology (Oftared), 28040 Madrid, Spain; rosa@ioba.med.uva.es; 4 Institute of Applied Ophthalmobiology (IOBA), Medical School, University of Valladolid, 47011 Valladolid, Spain; 5 ES Retina Asturias Association, 33212 Gijón, Spain; delgado-fernandez@hotmail.com; 6 San Vicente del Raspeig Campus, Ramón Margalef Institute, University of Alicante, 03690 Alicante, Spain

**Keywords:** capillary dropout, choroidal dystrophies, microaneurysms, optical coherence tomography, optical coherence tomography angiography, outer hyperreflective bands, vascular loops

## Abstract

Choroidal dystrophies comprise a group of chorioretinal degenerations. However, the different findings observed among these patients make it difficult to establish a correct clinical diagnosis. The objective of this study was to characterize new clinical findings by optical coherence tomography (OCT) and optical coherence tomography angiography (OCTA) in these patients. Four family members with a *PRPH2* gene mutation (p.Arg195Leu) were included. OCT was performed at the macula, and the thickness of the outer and inner retina, total retina, and choroid was measured. The features of the vascular network were analyzed by OCTA. Patients showed a decreased outer nuclear layer in the avascular area compared with the controls. Two patients presented greater foveal and parafoveal degeneration of the outer retina, whereas the most degenerated area in the rest was the perifovea. Disruption of the third outer band at the foveola is one of the first-altered outer bands. Slow blood flow areas or capillary dropout were main signs in the deep capillary plexus. Microaneurysms were frequently observed in less degenerated retinas. Vascular loops and intraretinal microvascular abnormalities (IRMAs) were present in the superficial plexus. Extensive degeneration of the choriocapillaris was detected. Phenotypic differences were found between patients: two showed central areolar choroidal dystrophy and the rest had extensive chorioretinal atrophy. These signs observed in OCT and OCTA can help to more appropriately define the clinical disease in patients with choroidal dystrophies.

## 1. Introduction

Choroidal dystrophies are known as ocular diseases that involve a degenerative process in the choroid vessels. However, mutations in the retinal pigment epithelium (RPE) [1] or in the photoreceptors are what lead to choroidal disorders. Among choroidal dystrophies, central areolar choroidal dystrophy (CACD), peripapillary choroidal dystrophy, and diffuse choroidal dystrophy [2], which is also called extensive chorioretinal atrophy (ECA), have been described. CACD is an inherited retinal dystrophy with a progressive degeneration of the macula, starting from middle age [3]. Classically, it was characterized by atrophy of the RPE and choriocapillaris, followed by a decrease in macular photoreceptors [4]. Consequently, this degeneration causes a gradual loss in visual acuity (VA) and a color perception deficiency between the second and fourth decades of life [5,6]. Four clinical stages are described for this pathology, ending with severe functional visual loss [5]. In autosomal-dominant CACD, the most frequent cause is *PRPH2* gene mutations [7]. The *PRPH2* gene encodes the *PRPH2* protein, a structural glycoprotein expressed in rods and cones, which is involved in the formation and maintenance of the outer segment (OS) discs [8]. Thus, mutations in this gene result in the first degeneration of photoreceptors [9].

Mutations in the *PRPH2* gene present not only large genetic heterogeneity but also marked phenotypic heterogeneity. Specific *PRPH2* gene mutations can cause different eye disorders among affected members of the same family [10,11]. Specifically, the c.584G > T, p.Arg195Leu mutation in the *PRPH2* gene has only been described in three families with CACD from Japan, Germany, and Spain [12,13,14,15]. The German family showed significant variability in the family phenotype with age [13]. Here, we describe new clinical findings using optical coherence tomography (OCT) and OCT angiography (OCTA) in four members from the Spanish family who showed phenotypic variability. The use of both techniques improved the capacity to differentiate among all clinical signs, allowing for classification of these patients with CACD or ECA.

## 2. Materials and Methods

### 2.1. Study Design and Initial Diagnosis

This was a cross-sectional study that included 4 members of a Spanish family with a mutation in the *PRPH2* gene (c.584G > T, p.Arg195Leu). The control group included 6 subjects (66.6% men, n = 4), with a mean age of 44 ± 5 years. The spherical refractive error of this group was between −1.5 and +2.0 diopters (D), cylinder was less than 1.0 D, and they had no previous ocular or systemic pathologies. Informed consent was obtained from each participant before performing the tests. The tenets of the Declaration of Helsinki and its subsequent amendments were followed, and the Ethics Committee on Human Research of the University of Alicante approved the study. OCT and OCTA images (Spectralis OCT, 6.9.4.0. version, Heidelberg Engineering Inc., Franklin, USA) were collected between June and December 2019 at the University of Alicante (Spain).

The medical history of each patient was gathered from when their visual symptoms began to present. Patient demographic data, age at symptom onset, best-corrected visual acuity (BCVA), Farnsworth Munsell D-15 test, and available visual field were assessed. Visual fields analyzed were performed with a standard automated perimetry, following SITA-Fast or SITA-Standard strategy (24-2 program and umbral test). Automated perimetry of visual field 10-2 (SITA-Fast or SITA-Standard strategy) results were discarded due to the low reliability of the test. Full field electroretinogram (ERG) recordings were also analyzed according to the International Society for Clinical Electrophysiology of Vision protocols [16].

Initial clinical diagnosis of these patients varied based on their genetic test, electroretinogram, and other functional tests such as visual evoked potential, visual fields, and fundus eye exams. Most of them were classified with retinal dystrophy or an atypical form of retinitis pigmentosa. Lastly, choroidal dystrophy was defined in at least 2 of them.

### 2.2. OCT and OCTA

All images were captured using Spectralis SD-OCT and an angiography module (version 6.9.4.0, Heidelberg Engineering Inc., Franklin, USA). Images were acquired under mesopic light conditions without pupillary dilation. A high-resolution (HR) horizontal profile at the foveola and a dense scan at the macula were obtained. Superficial vascular plexus (SVP), intermediate capillary plexus (ICP), deep capillary plexus (DCP), choriocapillaris, and choroid images were collected from OCTA images acquired in the macular area. The automated segmentation was corrected manually due to segmentation errors in OCT and OCTA images. Qualitative and quantitative (Figure S1) analyses with ImageJ 1.52n and Angio-tool software (0.6a version, National Cancer Institute, Bethesda, MD, USA [17]) were performed.

## 3. Results

### 3.1. Pedigree of the Spanish Family with the PRPH2 Gene Mutation

**Figure 1:**
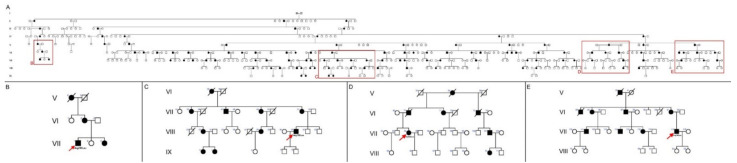
Entire pedigree of the Spanish family from 1750 to present. (**A**) Family members affected are indicated by black-filled shapes: 119 relatives were found to have retinal problems or blindness, of which 43.47% were women. A total of 60 of them are still alive and it is suspected that 60 more people in this family may also be affected. Most of these family members reside in the center of Cantabria, a province in Northern Spain. The 4 study family members within this pedigree are situated using red squares. (**B**–**E**) Pedigrees of the 4 study subjects. These subjects belong to different branches, though two of them are more closely related since they share a common descendant in the IV generation. A total of 3 patients (VII.1, VII.94, and VII.118) belong to the VII generation and the other (VIII.31) to the VIII generation. The autosomal dominant inheritance pattern can be clearly observed.

Mutation in the *PRPH2* gene (c.584G > T, p.Arg195Leu) was confirmed in these four family members. Due to the autosomal dominant inheritance pattern of the disease, other family members are also affected. The entire pedigree was constructed up to eleven generations ago, finding the first affected around 1750 (Figure 1 only shows up to nine generations). All subsequently affected members descended from this common ancestor (Figure 1A–E).

### 3.2. Clinical Findings and Evolution of the Disease

The study included four patients with a peripherin mutation (VII.1, VIII.31, VII.94, and VII.118), aged between 42 and 57 years old, and six control subjects. At the first ophthalmological examination, patients VIII.31, VII.118, and VII.1 had a similar VA in the worst eye (20/50 to 20/40). However, the decrease in VA during the progression of the disease varied considerably amongst them. Focusing on the eye with the BCVA during the follow-up period, VIII.31 and VII.1 showed a mean decrease in VA of 42.5% over 6.5 years, whereas VII.94′s VA only decreased by 22.5% over 14 years. Visual field indexes displayed that VII.94 had a marked central scotoma, whereas VII.118 and VII.1 presented wider-spread degeneration extending to the mid-periphery (Table 1). The results obtained by ERG showed alterations in retinal function, mainly in the photopic response (Table 1).

Farnsworth Munsell D-15 test results demonstrated a blue-yellow color vision defect (tritanopic defect) in VIII.31 and VII.94, and color blindness in VII.118 and VII.1.

Considering previous structural and functional eye examinations, VIII.31 and VII.94 presented atrophic changes restricted to the macular area that started in middle age. VII.118 and VII.1 showed large atrophic retinal areas involving the macula and mid-periphery, a large central scotoma in the visual field, and an important impairment of the photopic and scotopic ERGs. The most recent infrared (IR) fundus images from these patients are shown in Figure S2.

### 3.3. Features of OCT in CACD Patients: Macular Study and Retinal Tomography

**Figure 2:**
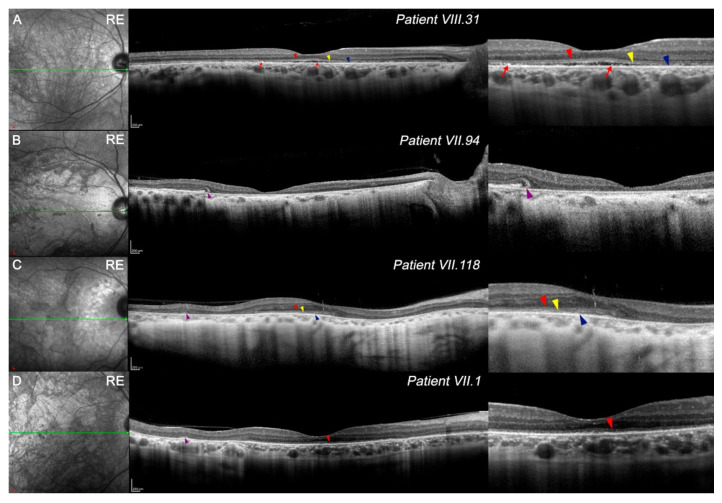
IR images (left) and OCT line scan at the foveola (right) in study patients. Right-side insets of all images show different signs in high magnification. (**A**) The retinal structure of patient VIII.31 is maintained, although a general thinning exists along the fovea, mainly on the temporal side. First (red arrowhead), second (yellow arrowhead), and fourth (blue arrowhead) outer retinal bands are preserved at some areas and some hyperreflective loci, which may be related to the third band (red arrows). (**B**) Patient VII.94 presented almost complete degeneration along the retina, specifically at the fovea, and outer tubulations can be observed (purple arrowhead). Part of the outer nuclear layer remained on the most temporal side. (**C**) Patient VII.118 showed a nasal and temporal reduction in thickness. Some outer retinal bands were observed at the fovea (red, yellow, and blue arrowheads), and outer tubulations related to this degenerative process can be also seen on the temporal side (purple arrowheads). (**D**) Patient VII.1 exhibited a marked reduction in the outer nuclear layer and only the first outer band is shown at the fovea (red arrowhead). Various outer tubulations were presented (purple arrowheads). Scale A–D: 200x 200 um. Images of contralateral eye presented a similar degeneration pattern, though are not shown. Red arrowhead, first outer retinal band; yellow arrowhead, second band; blue arrowhead, fourth band; red arrow, hyperreflective loci from band 3; purple arrowheads, outer tubulations.

Three-dimensional reconstructions of OCT images showed that VIII.31 had a retinal topographic pattern similar to a healthy retina (Figure S3A) whereas VII.94 showed a higher atrophy of the central retina (Figure S3B), and VII.118 (Figure S3C) had a better-preserved central area surrounded by atrophy in the mid-periphery area. OCT images at the foveola revealed foveal cavitation or optical gap in VIII.31 (Figure 2A). Quantitatively, all patients showed a marked decrease in total retinal thickness in the foveal avascular zone (FAZ) compared with the control group (Table 2); this reduction was greater in patients with lower VA.

Regarding the four outer retinal bands, only VIII.31 (Figure 2A) showed a slightly preserved external limiting membrane (ELM; red arrowhead), cone ellipsoid zone (EZ; yellow arrowhead), and the fourth band (RPEmitZ; blue arrowhead) at the foveola. As a foveal cavitation existed, the third band (OS tips phagocytosed by the RPE, PhaZ) was disrupted. Some hyperreflective loci could be related to the remnants of the third band (Figure 2A, red arrows). To a lesser extent, the first, second, and fourth outer retinal bands were relatively maintained at the foveola in VII.118 (Figure 2C, red, yellow, and blue arrowheads, respectively). Otherwise, the first outer band was still observed in some parts of the foveola in VII.1 (Figure 2D, red arrowheads). Some outer retinal tubulations were observed in VII.94, VII.118, and VII.1 (Figure 2B–D, purple arrowheads).

The outer retinal layers from FAZ limits in the four members were reduced compared with healthy controls (Table 2); however, differences in retinal degeneration were found among them. Although VIII.31 still had homogeneous degeneration throughout the retina (Figure 2, Table 2), the OCT images of VII.94 showed a more aggressive thinning between the foveola and parafoveal limits (Figure 2B, Table 2). In VII.118 and VII.1, perifoveal thinning of the outer retina seemed to be more pronounced than in the parafovea (Figure 2C,D, Table 2). The high signal transmission on OCT, observed mainly in patient VII.94 (Figure 2C,D), may imply a greater degeneration of the RPE.

The structure and organization of the inner retina was maintained (Figure 2), though its thickness was usually reduced compared with healthy patients (Table 2). Foveal and parafoveal degeneration of these layers was greater in VIII.31 and VII.94 than in VII.118 and VII.1 (Table 2). Most areas of the inner retina of VII.1 exceeded the retinal thickness of control patients. Ganglion cell layer (GCL) distribution can be observed in Figure S4.

In general, OCT images revealed a decrease in choroid thickness in all patients compared with heathy ones. Specifically, VII.94 and VII.118 showed more pronounced thinning in the nasal area (Table 2).

### 3.4. OCTA in PRPH2 Gene Mutation Patients

OCTA images centered on macula were analyzed, except VII.94, which was due to a lack of eye fixation.

#### 3.4.1. Quantitative and Qualitative Differences Found at SVP among Patients

**Figure 3:**
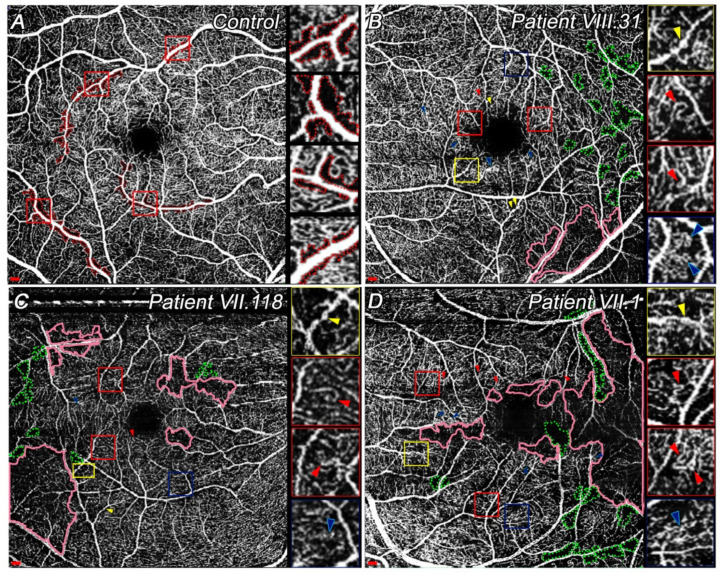
OCTA images of SVP centered at the macula in control and study patients. Insets at the right side of all images show different vascular signs in high magnification. (**A**) Superficial vessels of a healthy patient, showing capillary-free zone next to the large arteries (red dotted areas) and the FAZ, which is constituted by a single-layer of capillaries. Insets display several capillary-free zones. (**B**) Some slow blood flow areas (pink solid areas) and impaired perfusion areas (green dotted areas) already observed in addition to microaneurysms (yellow arrowheads), vascular loops or capillary bends (red arrowheads), and IRMAs (blue arrowheads) in patient VIII.31 (LE). (**C**) Similar signs to image (**B**) were detected in VII.118 (RE). (**D**) Larger slow blood flow areas (pink solid areas) and a greater number of capillary bends (red arrowheads) are shown. Capillary dropout areas (green dotted areas), microaneurysms (yellow arrowheads), and IRMAs (blue arrowheads) were also present in VII.1 (LE). Scale: 200 μm. Abbreviations: superficial vascular plexus, SVP; left eye, LE; right eye, RE. Yellow squares show microaneurysms; red squares indicate capillary bends or vascular loops; blue squares show IRMAs.

Patient VIII.31 had the largest avascular area in SVP compared with the other patients; however, his vessel density was greater (Table 3). In all study patients, total vessel length and average vessel length were lower (Figure 3A–D; Table 3). Lacunarity measurements revealed that VII.1. had a higher vascular alteration in SVP compared with other affected family members.

Qualitatively, VII.1 had a widespread area with blood flow reduction, whereas VIII.31 showed the smallest area affected amongst them (Figure 3B–D, pink areas). In all cases, these slow blood flow areas were mainly located on the temporal side, although VII.118 and VII.1 also had some vascular degeneration closer to the fovea (Figure 3C,D, pink areas). Total degeneration of these capillaries results in capillary dropout. It seems that capillary loss appears close to the large vessels, thus increasing the size of capillary-free zones (Figure 3B–D, green dotted areas). Moreover, microaneurysms (Figure 3B–D, yellow arrowheads), vascular loops or capillary bends (Figure 3B–D, red arrowheads), and intraretinal microvascular abnormalities (IRMA) (Figure 3B–D, blue arrowheads) were detected. Since OCTA image quality was insufficient, no difference was observed between vascular loops and capillary bends. All these patients had at least one of these signs in their SVP. Microaneurysms were more easily detectable in VIII.31 (Figure 3B, yellow arrowheads). Conversely, more vascular loops or capillary bends were found in a more degenerate vascular network in VII.1 (Figure 3D, red arrowheads). Although VII.1 and VII.31 had different degenerative processes, the IRMAs appeared similar (Figure 3B,D, blue arrowheads). The smallest number of these signs was detected in VII.118 (Figure 3C).

#### 3.4.2. Differences in ICP and DCP among Choroidal Dystrophies Patients Compared with Controls

**Figure 4:**
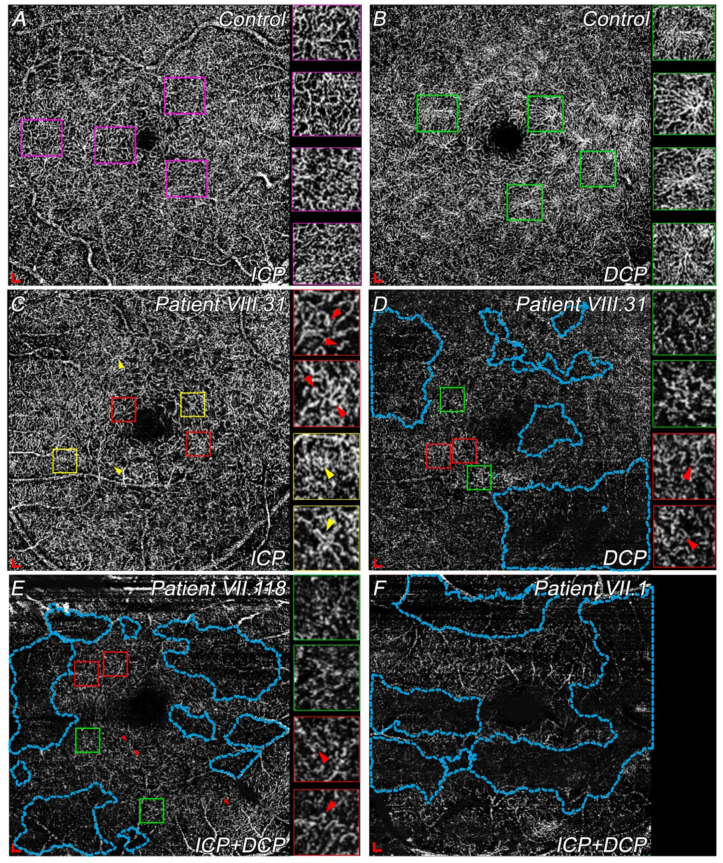
OCTA images of ICP and DCP centered at the macula in controls and patients with choroidal dystrophies. Insets at the right side of B, C, D, and E show different vascular signs in high magnification. (**A**) ICP of a healthy patient. Insets display the typical vessels with tortuosity. (**B**) DCP of a control subject where spider-like (green squares) structures surrounding the fovea can be seen. (**C**) ICP of patient VII.31 (LE) showing IRMAs (yellow arrowheads) and some capillary bends (red arrowheads). (**D**) Capillary dropout or slow blood flow areas (blue areas) with some degenerate spider-like structures (green squares) and capillary bends (red arrowheads), observed in the DCP of patient VII.31 (LE). (**E**) ICP and DCP images of VII.118 (RE), where larger areas of impaired vascular perfusion (blue areas) were detected in addition to some altered spider-like structures (green square) and capillary bends (red arrowheads). (**F**) Single slab of ICP and DCP of VII.1 (LE) showing widespread capillary dropout areas (blue areas). No spider-like structures were detected. Scale: 200 × 200 μm. Abbreviations: intermediate capillary plexus, ICP; deep capillary plexus, DCP; left eye, LE; right eye, RE. Green squares show spider-like structures; red squares indicate capillary bends or vascular loops; blue squares show IRMA.

During the degenerative process, the ICP loses its planar structure and simply allows a connection between the SVP and DCP. Thus, ICP and DCP images are shown in a single slab in VII.118 and VII.1 in Figure 4.

All patients showed a larger FAZ compared with controls (Table 3). The ICP of VIII.31 was quite preserved compared with a normal ICP (Figure 4A,C). The most remarkable difference between controls and VIII.31 was the larger size of the FAZ in the patient with the disease (Figure 4A,C; Table 3). Moreover, several vascular loops (Figure 4C, red arrowheads) and IRMAs (Figure 4C, yellow arrowheads) were present. Impaired perfusion or slow blood flow were easily detectable in VIII.31 and VII.118. However, OCTA image quality made it difficult to precisely differentiate between both signs (Figure 4D,E, the most-affected areas are indicated in blue dotted areas). Considering these signs, and although these patients seemed to be in different stages, the total extension of impaired vascular perfusion areas was similar among them (Figure 4D,E, blue dotted areas). Some capillary bends or vascular loops were also detected in VIII.31 and VII.118 (Figure 4D,E, red arrowheads) at the ICP and DCP. Most of the deep vascular plexus (ICP + DCP) in VII.1 had already degenerated (Figure 4F, blue dotted areas). Finally, a few spider-like structures were detected in VIII.31 and VII.118, and their size and extent were greatly reduced (Figure 4D,E, green square) compared with the large number of these structures present in healthy subjects (Figure 4B, some examples are marked in green). No spider-like structures were seen in VII.1 (Figure 4F).

#### 3.4.3. Choriocapillaris and Choroid Degeneration in CACD and ECA Patients

**Figure 5:**
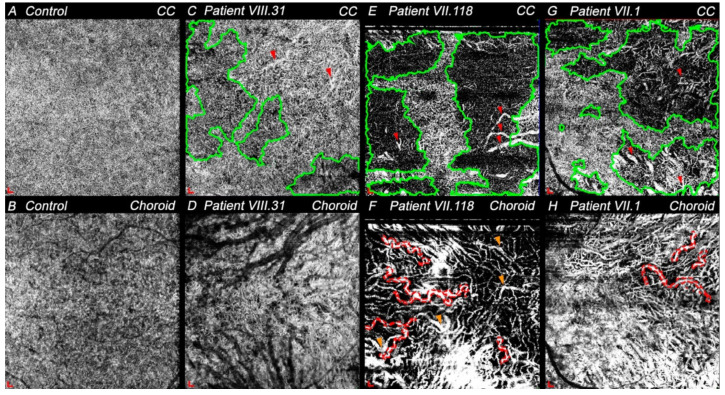
OCTA images of choriocapillaris (CC) and choroid centered at the macula in control and study patients. (**A**) Granular pattern typical of CC of a healthy patient. (**B**) Choroid seen with OCTA in a normal patient. Projection artifact of large retinal vessels can be observed. (**C**) CC from patient VIII.31 (LE) with attenuated areas (green areas) between the fovea and optic nerve. Red arrowheads indicate some inner choroid vessels due to the CC thinning. (**D**) Some inner and outer choroid vessels (dark vessels) can be observed in patient VIII.31′s choroid (LE). (**E**) The granular pattern of the CC is mainly preserved close to the fovea of VII.118 (RE). Green areas show impaired vascular perfusion and red arrowheads point to some choroid vessels. (**F**) Vascular tortuosity (red dotted areas) of larger vessels and focal vascular dilation (orange arrowheads) were seen in patient VII.118 (RE). (**G**) Larger impaired vascular perfusion areas (green areas) are shown in CC of VII.1 (LE). Thus, inner and outer choroid vessels were detected (red arrowheads). (**H**) Vascular tortuosity (red dotted areas) was present in VII.1 (LE) and inner choroid vessels were easily detected. Scale: 200 × 200 μm. Abbreviations: choriocapillaris—CC; left eye—LE; right eye—RE.

Since these diseases eventually involve choroid degeneration, the choriocapillaris and choroid were studied. The granular pattern of choriocapillaris typical of the center retina in healthy subjects (Figure 5A) was not observed in any of these patients (Figure 5C,E,G). VIII.31 showed the most similar choriocapillaris pattern to the controls, but relatively widespread signal voids areas were appreciable (Figure 5C, green areas). In addition, choriocapillaris thinning allowed the observation of some medium-sized vessels of the inner choroid (Figure 5C, red arrowheads). Larger areas of choriocapillaris dropout or signal voids were clearly visible in both VII.118 and VII.1 (Figure 5E,G, green areas). In them, a small central zone of choriocapillaris close to the fovea was preserved in VII.118, whereas choriocapillaris between the fovea and optic nerve in VII.1 maintained the granular pattern. Some medium- and large-sized choroidal vessels were detected (Figure 5E,G, red arrowheads).

In healthy patients, the choriocapillaris and RPE are preserved, which makes visualization of the choroid difficult by OCTA. Thus, little information could be obtained from these images (Figure 5B). However, in all patients with retinal degeneration where both layers were degenerating, it was possible to differentiate between inner and outer choroids (dark vessels). Choriocapillaris and RPE were partly preserved in VIII.31, which made it possible to visualize only some of the inner and outer choroid vessels (Figure 5D). OCTA images from choroid of VII.118 and VII.1 revealed that both individuals presented vascular tortuosity, mainly in the larger vessels (Figure 5F,H, red dotted areas), and the radial distribution pattern from the fovea of the outer choroid was observed. The choroid of these patients also showed areas with lower vascular density (Figure 5F,H). Specifically, VII.118 had more impaired perfusion areas and fewer vessels, with most of them being narrower compared with VII.1. In addition, focal vascular dilation was noted in VII.118 (Figure 5F, orange arrowheads). Thus, considering these parameters, the inner choroid seemed more preserved in VII.1 than in VII.118.

## 4. Discussion

Here, we describe the clinical findings over time in four members of a Spanish family with the p.Arg195Leu mutation in the *PRPH2* gene, and who were diagnosed with central choroidal degenerations. Qualitative and quantitative analyses of OCT and OCTA images were performed to understand the retinal findings observed during the degenerative process. Retinal disease was detected from the third decade of life in all of them, as also described in other studies about this mutation [12,13] and other mutations in *PRPH2* [6,18,19]. However, this specific mutation was confirmed only a few years after the first examination in three of them (VIII.31, VII.118, and VII.1), and 20 years afterwards in VII.94. Despite the age of onset of visual problems being similar in all four, the progression of the retinal degeneration seemed to differ. Unlike Keilhauer et al., who found a change in the CACD phenotype with age [13], we already found a different phenotype in younger subjects of this Spanish family with the *PRPH2* gene mutation. That is, according to the acquired OCT and OCTA images and the medical history gathered, VIII.31 and VII.94 presented CACD, whereas VII.118 and VII.1 showed a different phenotype: extensive chorioretinal atrophy (ECA).

This difference in phenotypes or variability in clinical signs in members of the same family are currently difficult to explain, though some studies have already tried to provide this explanation for other *PRPH2* gene mutations [13,20]. In our study, clear clinical differences related to VA and visual field were observed between CACD and ECA patients. These variations can be easily explained because CACD induces higher central retinal degeneration, affecting the maximum VA zone, whereas ECA initially affects the mid-peripheral retina. Despite these clinical differences, findings over time showed that the disease progression was faster when the patient had an intermediate stage and higher VA (VIII.31 and VII.1), and stabilized when a later stage existed presenting low VA (VII.94). This could be explained by both diseases having their degeneration peak at early–intermediate stages, as occurs in other retinal dystrophies [21]. It is remarkable that most of them experienced color vision problems from the age of 20. The main color defect detected later was tritanopia, involving blue cones [13], which are located in the parafovea [22]. This correlates with the initially aggressive degeneration in this area.

Considering the previous classification [5] and our results with OCT and OCTA imaging, stages of the disease are defined for these four patients. Specifically, IR, OCT, and OCTA images in CACD patients suggest VII.94 was at a later stage than VIII.31 and appeared to show an age-dependent progression. Otherwise, this age-dependent progression did not seem to be adjusted in our ECA patients: structural tests in VII.1 showed that he presented a more advanced stage than VII.118, even though the first patient is younger. Greater foveal sparing was observed in VII.118; thus, his VA was higher.

Classically, CACD has been defined as an atrophy of the RPE and the choriocapillaris, where photoreceptor degeneration cause a large loss of central vision [4,5,6]. Choriocapillaris and choroid degeneration were also thought to be one of the first events occurring during this dystrophy, along with degeneration of the RPE, since vascular alterations were found in the choroid with fluorescein angiography and indocyanine green angiography [23,24,25,26,27,28]. Nevertheless, considering our results with OCT and OCTA images, we propose a redefinition of the disease, regarding CACD as a degenerative disease of the retina where photoreceptors, mainly cones, are the first affected, causing RPE and choriocapillaris degeneration. Something similar could be applied for ECA, showing a more widespread alteration, and affecting both types of photoreceptors. Thus, both diseases could be categorized as mainly cone dystrophies, at least when caused by the p.Arg195Leu *PRPH2* mutation.

Some correlation appears to exist between the reduction in retinal thickness in the FAZ and the loss of VA, which could be due to a decrease in cone photoreceptors at the ONL. In addition, foveal cavitation, a sign of cone dysfunction described in other retinal dystrophies [7,29,30], was present in VIII.31. Additionally, foveal cavitation was accompanied by the disruption of the third outer retinal band (PhaZ) in this patient, indicating that OS phagocytosis by RPE was impaired [31,32]. Considering the four outer retinal bands, OCT images revealed that the degeneration process appears similar to other pathologies [33,34]. The finding that *PRPH2* protein is present in the OS discs of photoreceptors [8] may explain why PhaZ disappears at first and ELM is the last to be disrupted. In these four cases, outer and inner retinal alterations did not show different degeneration on either the nasal or temporal side compared with the controls. However, outer retinal degeneration and vascular disturbances seemed to be more aggressive on the temporal side, which agrees with published studies using experimental models of other retinal dystrophies [35,36,37].

In addition to retinal degeneration, vascular disturbances such as an increase in the size of the avascular zone at the SVP or DCP are appreciable in these patients. Specifically, the larger avascular area in the SVP in the CACD patient may be explained by his own degeneration pattern. However, the greater size of the FAZ of VII.1 compared with CACD might be associated with his later-stage disease and, thus, his lower VA. The density and length of the superficial vessels in CACD and ECA patients were reduced compared with controls, agreeing with studies of other retinal diseases [38,39,40]. Some studies established a positive correlation between macular function and vessels’ length and density [38,41]; however, in ECA patients, a reduction in VA might not be associated with a lesser vascular density if foveal sparing exists until later stages. Otherwise, a lower vessel density may imply greater visual field loss in this mutation.

Vascular disturbances are thought to lead to decreased blood flow velocity in the capillaries [42], which could be seen as slow blood flow areas by OCTA. Microaneurysms appear to be more frequent at earlier stages of the disease. As they are proper signs of vascular degeneration onset or the less severe forms [43], their presence may indicate higher risk of progression to more severe forms of the disease, as occurs in other pathologies [44]. Vascular loops or capillary bends and IRMAs, which are proper signs of vascular disorders [42,45,46], were similarly appreciable in these patients. Nonetheless, widespread impaired vascular perfusion areas were more numerous in later stages (VII.1), which indicated a lower vascular density. All these findings show that, although there are fewer vessels in these late stages, most have some vascular alteration proportional to earlier stages, so the final vascular disturbance is greater. The DCP in these dystrophies is probably, and usually, the most-disturbed plexus, since it is closest to affected photoreceptors, as has been recently described in retinitis pigmentosa [47,48]. Our results showed that DCP, SVP, and choriocapillaris degenerated areas seems to be linked; thus, the lack of vessels is not associated with low-quality images.

In this study, the obtained results allowed us to redefine the clinical disease, although only four patients were analyzed. Moreover, new clinical findings were described for the first time in OCT and OCTA for these choroidal dystrophies. Thus, this study’s findings encourage characterizing the rest of the affected family members to facilitate a clinical diagnosis. Despite the high quality of the acquired OCTA images, these can be affected by small artifacts associated with poor eye fixation in this kind of patient. Furthermore, the quantitative analysis of OCT and OCTA images was performed by external software, which could have conditioned the results.

## 5. Conclusions

Considering the previous information and the clinical findings of these patients, we reach the following conclusions: First, the outer nuclear layer thickness and the presence of four outer retinal bands at the foveola and the FAZ size are the signs that most affect VA. The parafoveal and perifoveal degeneration of the outer and inner retina or other vascular alterations seem not to be as decisive in VA. Second, this *PRPH2* mutation firstly causes alterations in the photoreceptor–RPE complex, followed by the degeneration of the inner retina, retinal plexuses, and choriocapillaris. Third, this study confirms that vascular alterations are signs of these choroidal dystrophies. Forth, despite all family members having the same mutation, there was wide variability in terms of clinical findings, progression of the disease, and visual acuity among patients. Fifth, OCT and OCTA images can help to more appropriately distinguish the clinical disease (CACD from ECA) by offering more detailed information than fundus images. Finally, all the affected members coming from a common ancestor allowed us to locate the disease in a specific area, with a large number of affected people, and, thus, enabled the development of clinical trials.

## Figures and Tables

**Table 1:** Clinical findings.

Patient/Sex	Age at Diagnosis	VA at Diagnosis		Current Age	Current VA		Visual Field Results		Age at ERG/ERG Results
		RE	LE		RE	LE	4–5 Years after Diagnosis (RE/LE) (dB)	12–15 Years after Diagnosis (RE/LE) (dB)	
VII.1/M	39	20/40	20/50	46	20/100	20/100	24-2 MD: −27.22/−26.25 PSD: 6.91/7.30 VFI: 16%/18%	–	39/Scotopic ERG reduced. Photopic responses decreased (absent in red light)
VIII.31/M	33	20/50^+2^	20/20^−2^	42	20/125^−1^	20/50	–	–	33/Scotopic ERG acceptable answer. Photopic responses notably reduced.
VII.94/F	33	20/100	20/63^−1^	57	20/125	20/200^−2^	24-2 MD: −14/−14.12 PSD: 13.6/14.06	24-2 MD: −17.81/−15.51 PSD: 11.77/10.81	37/Scotopic ERG with a minimal reduction in amplitude. Photopic ERG and Flicker highly reduced.
VII.118/M	37	20/40^+1^	20/32^+3^	50	20/63	20/25	–	24-2 MD: −28.58/−28.49 VFI: 10%/11.5%	37/Scotopic ERG with a reduction in amplitude and latencies. Photopic and Flicker ERG absent.

Abbreviations: VA—visual acuity; RE—right eye; LE—left eye; ERG—electroretinogram.

**Table 2:** Thickness in total, outer, and inner retina, and choroid in optical coherence tomography (OCT).

Patients	Total Retinal Thickness (µm)	Outer Retina (µm)				Inner Retina (µm)		Choroid (µm)	
	Measurements at FAZ	From FAZ limits (500 µm) to 1.2 mm (Parafovea Limits)		From 1.2 mm to 2.8 mm (Perifovea)		From FAZ Limits (500 um) to 2.8 mm (Perifovea Limits)		From Foveola (0 um) to 2.8 mm (Perifovea Limits)	
		Temporal	Nasal	Temporal	Nasal	Temporal	Nasal	Temporal	Nasal
Control group	230 ± 20	194 ± 8	194 ± 7	150 ± 10	160 ± 10	141 ± 6	165 ± 9	280 ± 40	260 ± 40
VIII.31	137.7 ± 0.5	94 ± 2	94 ± 1	87 ± 2	98 ± 7	130 ± 10	150 ± 6	230 ± 60	290 ± 50
VII.94	20 ± 20	4 ± 5	13 ± 2	40 ± 20	4 ± 8	70 ± 10	90 ± 10	230 ± 50	120 ± 50
VII.118	173 ± 5	120 ± 10	30 ± 30	30 ± 30	20 ± 20	110 ± 20	145 ± 9	140 ± 10	140 ± 20
VII.1	119 ± 2	89 ± 4	90 ± 10	40 ± 30	50 ± 10	140 ± 20	210 ± 20	240 ± 20	220 ± 30

Retinal thickness at the FAZ shows both eyes’ average for the study patients. The mean values of the control group (n = 6) correspond to one eye of each. Only the right eye is shown to simplify data for outer and inner retina and choroid. Abbreviations: FAZ—foveal avascular zone; RE—right eye; LE—left eye; SD—standard deviation.

**Table 3:** Quantitative analysis of retinal plexuses.

Patients	SVP Parameters							ICP-DCP Parameters		
	Avascular Area (mm^2^)			Angio-Tool Measurements				FAZ (mm^2^)		
	RE	LE	Mean ± SD	Vessel Density (% Vessels)	Total Vessels Length (mm)	Average Vessels Length (mm)	Mean Lacunarity (Λ)	RE	LE	Mean ± SD
Control group	0.4 ± 0.1	0.4 ± 0.2	0.370 ± 0.001	53 ± 2	530 ± 30	10 ± 10	0.022 ± 0.003	0.3 ± 0.1	0.3 ± 0.1	0.25 ± 0.01
VIII.31	0.61	0.63	0.62 ± 0.01	47 ± 3	504 ± 7	4.0 ± 0.4	0.040 ± 0.003	0.39	0.47	0.43 ± 0.06
VII.118	0.54	0.34	0.4 ± 0.1	39.4 ± 0.7	450 ± 10	1.6 ± 0.5	0.049 ± 0.001	0.42	0.32	0.37 ± 0.07
VII.1	0.57	0.58	0.575 ± 0.007	41 ± 2	430 ± 30	2.40 ± 0.04	0.057 ± 0.001	0.46	0.47	0.465 ± 0.007

One measurement was performed on the avascular area per each OCT angiography (OCTA) image. Angio-tool values corresponds to the mean ± SD among both eyes for study patients. Mean values of the control group (n = 6) correspond to one eye of each. Abbreviations: SVP—superficial vascular plexus; RE—right eye; LE—left eye; SD—standard deviation.

## Data Availability

The data presented in this study are available in request from the corresponding author.

## Supplementary Materials

The following are available online at https://www.mdpi.com/article/10.3390/diagnostics11050777/s1, Figure S1: Location of macular components in OCT images; Figure S2: Infrared images of right (upper) and left (down) eyes of control (A,B) and patients with *PRPH2* gene mutation (C-I); Figure S3: Three-dimensional reconstruction from a dense OCT scan of some study patients associated to p.Arg195Leu mutation; Figure S4: Volume scan of the GCL thickness map (right) at 1, 2, and 3 circle diameters from the foveola. References [32,42,45,46,49,50] are cited in the Supplementary Materials.
