# Supplementary material for: Phenotypic Differences in a PRPH2 Mutation in Members of the Same Family Assessed with OCT and OCTA
Source: Diagnostics (Basel). 2021 Apr 26;11(5):777. doi: 10.3390/diagnostics11050777 (PMC8146155; doi:10.3390/diagnostics11050777)
Supplement: Supplementary file 1 [file diagnostics-11-00777-s001.zip › diagnostics-1182627-SI.pdf]

diagnostics

MDPI

Supplementary Material

Phenotypic Differences in a *PRPH2* Mutation in Members of the Same Family Assessed with OCT and OCTA

Henar Albertos-Arranz [1,†], Xavier Sánchez-Sáez [1,†], Natalia Martínez-Gil [1], Isabel Pinilla [2,3], Rosa M. Coco-Martin [3,4], Jesús Delgado [5] and Nicolás Cuenca [1,3,6*]

1 Department of Physiology, Genetics and Microbiology, University of Alicante, 03690 Alicante, Spain; henar.albertos@ua.es (H.A.-A.); xsanchez@ua.es (X.S.-S.); natalia.martinez.gil@ua.es (N.M.-G.); cuenca@ua.es (N.C.)
2 Department of Ophthalmology, Lozano Blesa University Hospital, Aragon Health Science Institute (IIS Aragón), 50009 Zaragoza, Spain; ipinilla@unizar.es (I.P)
3 National Institute of Health Carlos III (ISCIII), (RETICS) Cooperative Health Network for Research in Ophthalmology (Oftared), 28040 Madrid, Spain
4 Institute of Applied Ophthalmobiology (IOBA), Medical School, University of Valladolid, 47011 Valladolid, Spain; rosa@ioba.med.uva.es
5 ES Retina Asturias Association, 33212, Gijón, Spain; delgado-fernandez@hotmail.com (J.D.)
6 Ramón Margalef Institute, San Vicente del Raspeig Campus, University of Alicante, 03690 Alicante, Spain
* Correspondence: cuenca@ua.es; Tel.: +34-965-909-916
† These authors contributed equally to this paper

METHODS.

Figure S1. Location of macular components in OCT images.

RESULTS.

Figure S2. Infrared images of right (upper) and left (down) eyes of control (A-B) and patients with *PRPH2* gene mutation (C-I).
Figure S3. Three-dimensional reconstruction from a dense OCT-scan of some study patients associated to p.Arg195Leu mutation.
Figure S4. Volume scan of the GCL thickness map (right) at 1, 2 and 3 circle diameters from foveola.

METHODS

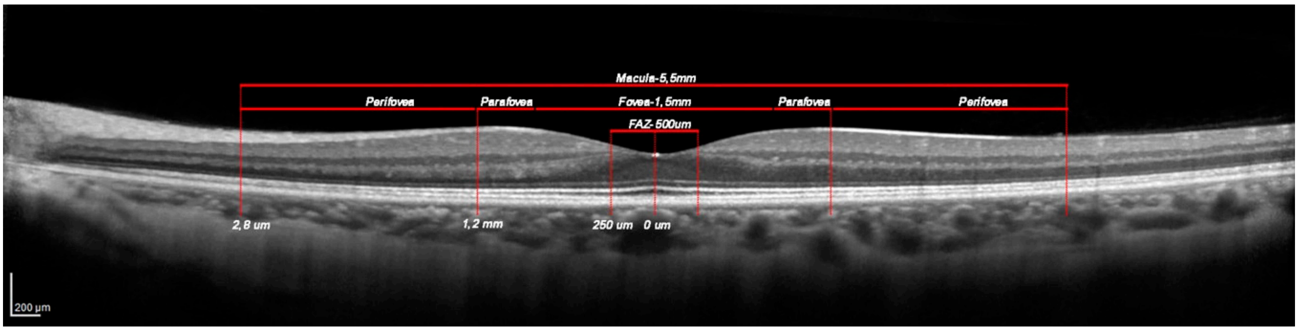

Figure S1. Location of macular components in OCT images. Macula has a diameter of 5.5 mm and includes perifovea, parafovea, fovea (1.5 mm of diameter) and foveal avascular zone (FAZ, 500 µm). Measurements were taken into account from foveola, whose location was considered as origin point (0 um).

A high-resolution (HR) horizontal profile at foveola (8.7 mm, 1536 A-scans) was acquired. Outer retina (inner and outer segment of photoreceptors, outer nuclear layer and outer plexiform layer), inner retina (from inner nuclear layer to retinal nerve fiber layer) and choroid were measured each 400 µm from the foveola (0 µm) to macula limits (Figure S1) when possible with ImageJ 1.52n (National Institutes of Health, Bethesda, Maryland,

USA). Differences in retinal degeneration along these OCT line scans obliged to cluster measurements in foveal avascular zone (FAZ), parafovea and perifovea (Figure S1). Moreover, a dense scan at the macula (20°×20°, 49 B-scan) to obtain a 3D reconstruction was carried out. Segmentation of retinal layers was performed from this dense scan to obtain ganglion cell layer volume map. Automated segmentation was corrected manually due to segmentation errors. Infrared (IR) fundus images were extracted from high resolution OCT images. The threshold for image quality was at least 25 over 40 dB.

OCTA images were performed at the macula with the high-speed (HS) mode (lateral resolution of 11µm/pix and 512 B-scans) and a field of view of 20°. Superficial vascular plexus (SVP), intermediate (ICP) and deep capillary plexuses (DCP), choriocapillaris and choroid images were collected from each test. After acquiring images, manual segmentation corrections were performed to avoid interpretation failures in OCTA images when possible. Specifically, these segmentation corrections were done considering histological localization of plexuses[1]. Thus, SVP slab comprised GCL thickness; ICP slab consisted of the innermost part of the inner nuclear layer (INL); and DCP contained a slab in the outermost part of the INL. Outer retina sometimes had to be added to DCP segmentation in order to include entirely this plexus. In addition, a single slab including ICP and DCP was obtained when separation between them was not possible. Two different slabs were obtained from choriocapillaris and inner and outer choroid. Overlapping effects between these two layers in images must be considered due to the degeneration itself.

Both eyes were imaged, and all these tests were carried out when it was possible. Therefore, in order to improve images quality and the signal-to-noise ratio, the automatic real-time [ART] mode was used trying to obtain the highest quality. However, number of frames of this ART-mode achieved for each final image depended on the fixation of each patient[2].

Once manual segmentation was performed, en face OCTA images were selected for each plexus and qualitative and quantitative analyses were carried out when possible.

Avascular zone was measured at SVP and where the vascular ring was formed with ImageJ 1.52n. Despite vascular ring of FAZ is at ICP level[1] , retinal degeneration of these patients made impossible to detect FAZ in this plexus. Thus, en face OCTA images with this vascular ring were obtained specifically to measure this area.

Angio tool (0.6a version, National Cancer Institute, Bethesda, Maryland, USA) allowed to analyze different parameters: vessel density (% vessels), total vessels length (mm), average vessels length (mm) and mean lacunarity (Λ). Lacunarity is a parameter related to the size and heterogeneity of lacunas or gaps present in the images(3). Mean lacunarity has a range between 0–1: the highest lacunarity, the greater number of gaps or lacunas in the image and the greater degeneration. Moreover, slow blood flow or impaired perfusion areas, microaneurysms, vascular loops or capillary bends, IRMA[4–6] and spider-like structures were also studied qualitatively. Spider-like structures are vascular patterns similar to a spider commonly found in DCP of healthy subjects[1].

RESULTS

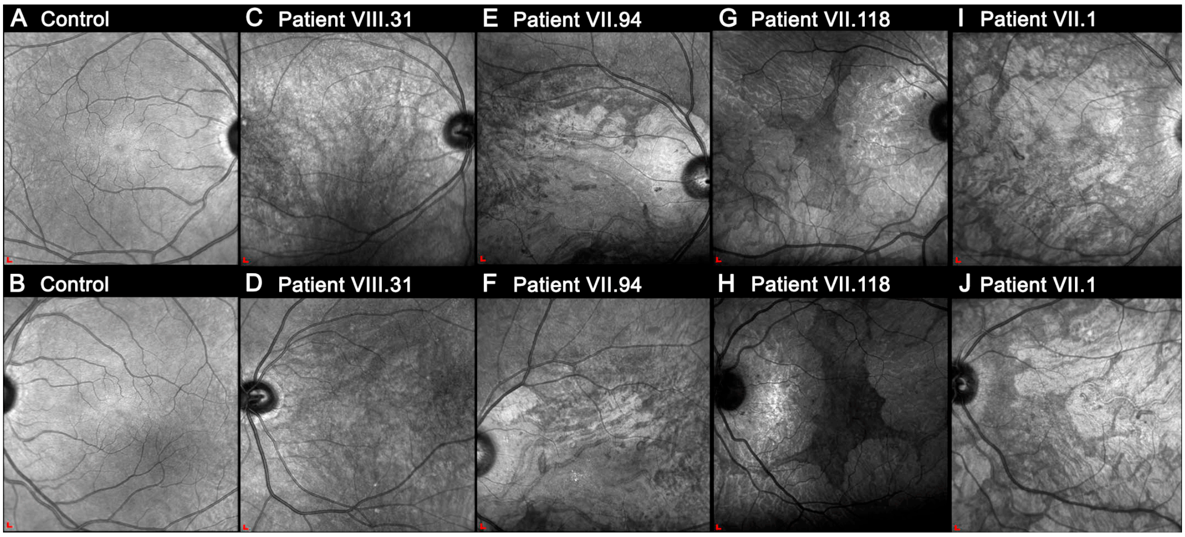

Figure S2. Infrared images of right (upper) and left (down) eyes of control (A-B) and patients with *PRPH2* gene mutation (C-I).(**A**–**B**) Fundus images of a healthy control. (**C**–**D**) Patient VIII.31 showed parafoveal hypopigmented areas with partial visualization of choroidal blood vessels without atrophic signs. Patient VII.94 (**E**–**F**) showed a large area of chorioretinal atrophy between vascular arcades, affecting the macular area. Patient VII.118 (**G**–**H**) had various well-circumscribed atrophy areas with hyperpigmented borders and foveal sparing while patient VII.118 showed affectation also around the central area (**I**–**J**). Choroidal blood vessels were visible in some areas in both cases. All of them showed a symmetric degeneration in both eyes. Scale A-J 200um (vertical and horizontal).

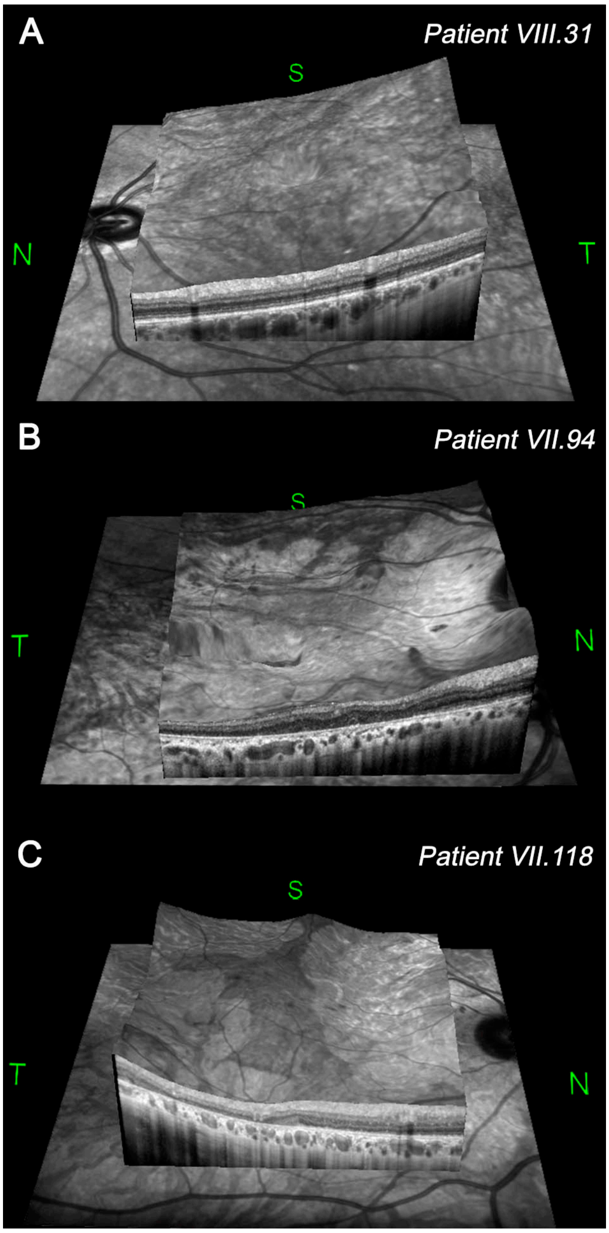

Figure S3. Three-dimensional reconstruction from a dense OCT-scan of some study patients associated to p.Arg195Leu mutation. (**A**) A retinal topography similar to a normal pattern is seen in patient VIII.31. (**B**) Complete alteration of macular topography exists in VII.94. (**C**) A parafoveal depression can be observed in VII.118.

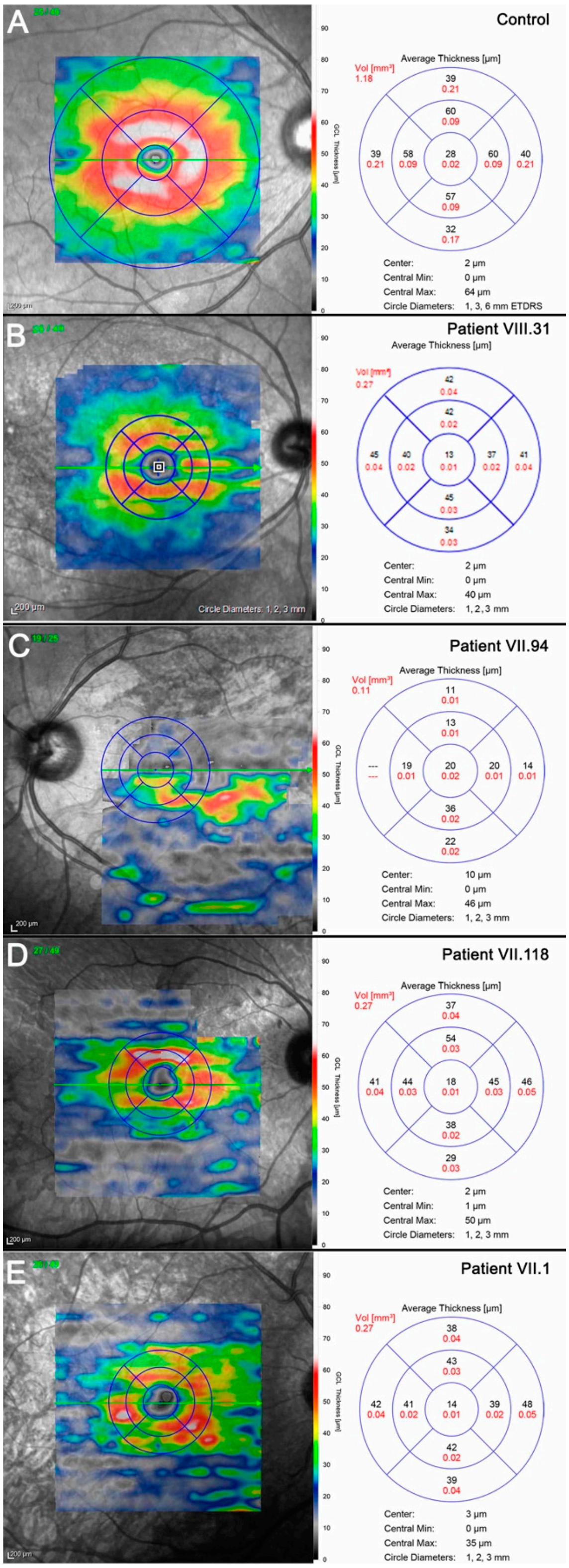

Figure S4. Volume scan of the GCL thickness map (right) at 1, 2 and 3 circle diameters from foveola. (**A**) Normal GCL distribution. (**B**, **C**) Color maps (left) show a clear reduction in the GCL thickness between patient VIII.31 (**B**) and VII.94 (**C**). The higher volume in the central circle of VII.94 might be associated to a re-distribution layer or segmentation errors. (**D**, **E**) GCL and the average thickness map of each quadrant of ECA patients show similar results among them due to the fact that a different cell distribution exists for each patient. Scale A–E: 200×200 μm². White and warm colors correspond with thicker areas while black and cold colors correspond with thinner areas.

References

1. Cuenca, N.; Ortuño-Lizarán, I.; Sánchez-Sáez, X.; Kutsyr, O.; Albertos-Arranz, H.; Fernández-Sánchez, L.; et al. Interpretation of OCT and OCTA images from a histological approach: Clinical and experimental implications. Prog Retin Eye Res 2020, 77(July 2019), 100828. Available from: https://doi.org/10.1016/j.preteyeres.2019.100828
2. Bax, N.M.; Valkenburg, D.; Lambertus, S.; Klevering, B.J.; Boon, C.J.F.; Holz, F.G.; et al. Foveal Sparing in Central Retinal Dystrophies. Invest Ophthalmol Vis Sci 2019, 60(10), 3456–67.
3. Gould, D.J.; Vadakkan, T.J.; Poché, R.A.; Dickinson, M.E. Multifractal and lacunarity analysis of microvascular morphology and remodeling. Microcirculation 2011, 18(2), 136—151. Available from: https://europepmc.org/articles/PMC3049800
4. Matsunaga, D.R.; Yi, J.J.; De Koo, L.O.; Ameri, H.; Puliafito, C.A.; Kashani, A.H. Optical coherence tomography angiography of diabetic retinopathy in human subjects. Ophthalmic Surg Lasers Imaging Retin 2015, 46(8), 796–805.
5. Mo, S.; Krawitz, B.; Efstathiadis, E.; Geyman, L.; Weitz, R.; Chui, T.Y.P.; et al. Imaging foveal microvasculature: Optical coherence tomography angiography versus adaptive optics scanning light ophthalmoscope fluorescein angiography. Investig Ophthalmol Vis Sci 2016, 57(9), OCT130–40.
6. Tam, J.; Dhamdhere, K.P.; Tiruveedhula, P.; Lujan, B.J.; Johnson, R.N. Jr.; et al. Subclinical Capillary Changes in Non Proliferative DiabeticRetinopathy 2012, 89(5), 692–703.
